# A guide for kernel generalized regression methods for genomic-enabled prediction

**DOI:** 10.1038/s41437-021-00412-1

**Published:** 2021-03-01

**Authors:** Abelardo Montesinos-López, Osval Antonio Montesinos-López, José Cricelio Montesinos-López, Carlos Alberto Flores-Cortes, Roberto de la Rosa, José Crossa

**Affiliations:** 1 Departamento de Matemáticas, Centro Universitario de Ciencias Exactas e Ingenierías (CUCEI), Universidad de Guadalajara, 44430 Guadalajara, Jalisco México; 2 Facultad de Telemática, Universidad de Colima, 28040 Colima, México; 3 Departamento de Estadística, Centro de Investigación en Matemáticas, 36023 Guanajuato, México; 4 Colegio de Postgraduados (CP), Campus Tabasco, Producción Agroalimentaria en el Trópico, H. Cárdenas, Tabasco México; 5 Colegio de Postgraduados, Campus Montecillos, CP 56230 Montecillos, Edo. de México México; 6 Biometrics and Statistics Unit, International Maize and Wheat Improvement Center (CIMMYT), Km 45, CP 52640 Carretera Mexico-Veracruz, México

**Keywords:** Genomics, Plant sciences

## Abstract

The primary objective of this paper is to provide a guide on implementing Bayesian generalized kernel regression methods for genomic prediction in the statistical software R. Such methods are quite efficient for capturing complex non-linear patterns that conventional linear regression models cannot. Furthermore, these methods are also powerful for leveraging environmental covariates, such as genotype × environment (G×E) prediction, among others. In this study we provide the building process of seven kernel methods: linear, polynomial, sigmoid, Gaussian, Exponential, Arc-cosine 1 and Arc-cosine L. Additionally, we highlight illustrative examples for implementing exact kernel methods for genomic prediction under a single-environment, a multi-environment and multi-trait framework, as well as for the implementation of sparse kernel methods under a multi-environment framework. These examples are followed by a discussion on the strengths and limitations of kernel methods and, subsequently by conclusions about the main contributions of this paper.

## Introduction

Genomic selection (GS) is a methodology that predicts the breeding (or genetic) values of candidate individuals using a statistical machine learning model trained with a reference (training) population for which only phenotypic and genotypic information is measured (Meuwissen et al. 2001). However, the quality of GS results are strongly dependent on the quality of both genotypic and phenotypic training data. For this reason, the genotypic information should be representative of the genome of each line, just as the lines in the training sample should also be representative of the target (testing set) for which predictions are required. Moreover, the statistical machine learning algorithms used for performing the predictions are of paramount importance since the assumptions of each of these models can either limit or improve the prediction performance in a particular data set. For example, linear regression models cannot capture non-linear patterns in input data since they presuppose only linear patterns. For example, Waldmann (2018) found that the resulting testing set mean square error (MSE) on the simulated TLMAS2010 data were 82.69, 88.42, and 89.22 for the multilayer-perceptron (MLP) non-linear model, the genomic best linear unbiased predictor (GBLUP) and Bayesian Lasso (BL) respectively. Additionally in this study, he found that the non-linear model (MLP) only outperformed the linear models by 1%. However, Waldmann et al. (2020) later used the same TLMAS2010 data and found that under the convolutional neural network (CNN) non-linear model, the MSE was equal to 62.34, while under linear models (GBLUP and BL), it produced a MSE over folds of 88.42 and 89.22, respectively. In another study, Khaki and Wang (2019) used a maize dataset of the 2018 Syngenta Crop Challenge to evaluate the prediction performance of the MLP (deep learning method) against the performance of Lasso regression (LR) and regression tree (RT). They reported that the MLP model with 20 hidden layers outperformed conventional genomic prediction models (LR and RT). Furthermore, Ma et al. (2018) also used CNN to predict phenotypes from genotypes in wheat, where they found that the non-linear method, CNN, outperformed the GBLUP method.

In plant breeding, many traits like grain yield have a complex genetic architecture that is not well understood (Golan and Rosset 2014) and when the modeling process is only able to capture linear patterns, it cannot perform the best possible mapping between the phenotypic and genotypic information. For this reason, the phenotypic prediction for such traits remains a major challenge. One of the strategies used to improve the phenotypic prediction is to consider the epistatic effects, within the predictor, that are genetic interactions (Cordell 2002). Epistatic interactions are not particular to plant breeding, and there is growing empirical evidence that they are also very common in other fields of biology (Moore and Williams 2009; Lehner 2011; Hemani et al. 2014; Buil et al. 2015). While epistatic interactions are biologically plausible on the one hand (Zuk et al. 2012), they are difficult to detect on the other (Cordell 2009), suggesting that they may be highly influential in our limited success at modeling complex heritable traits.

Martini et al. (2020) demonstrated that the whole genome epistasis models with interactions between different loci could be approximated based on Hadamard powers of the additive genomic relationship and provided an explicit formula to quantify the approximate error. The authors shown that the quality of the given approximation decreases when the degree of the interaction increases. Jiang and Reif (2015) found that modeling epistasis explicitly in the linear predictor of the genomic prediction model increases prediction performance, which can also be captured by using a Gaussian Kernel. Furthermore, these authors also point out that the prediction performance can be improved by modeling epistasis for selfing species, with the exception of outcrossing species. Another common approach for modeling epistatic effects is to include the main line effects with the additive genomic relationship matrix (GRM) as a correlation matrix, with the epistatic relationship matrix computed as the Hadamard product of the additive relationship matrix by itself (Henderson 1985). There is empirical evidence that when the dominant or epistatic effects are modeled in addition to the additive effects, simulation studies show up to 17% more accurate predictions based on the sum of both, as opposed to predictions based solely on additive effects (Wellmann and Bennewitz 2012; Da et al. 2014).

For this reason, kernel regression methods like Reproducing Kernel Hilbert Spaces (RKHS) regression have been proposed in plant and animal breeding to capture complex, non-linear patterns (Gianola et al. 2006; Gianola and van Kaam 2008). The basic idea of RKHS methods is to project the original independent variables given in a finite dimensional vector space into an infinite-dimensional Hilbert space. Kernel methods transform the independent variables (inputs) using a kernel function, followed by the application of conventional machine learning techniques to the transformed data to achieve better results. RKHS methods based on implicit transformations have become very popular for analyzing non-linear patterns in data sets from various fields of study. Kernel methods obtain measures of similarity between objects that do not have a natural vector representation. Even though kernel methods exploit complexity to improve prediction accuracy, they do very little to increase the understanding of the complexity since parameters like beta coefficients in linear regression are difficult to interpret. Heritability can also be estimated with kernel methods under a mixed model framework—albeit this only works with some types of kernels (Mathew et al. 2018; Ma and Dicker 2019). On the other hand, three crucial advantages of kernel methods over deep neural networks are that (a) these methods guarantee a global minimum (unique solution), (b) are considerably easier to tune since they have few hyper-parameters and (c) take advantage of the so-called kernel trick to capture non-linear patterns at a reasonable computational cost, working efficiently in the context of large independent variables (*p*) and small sample size (*n*) (Morota et al. 2013).

In general, the building process of kernel machines consists of two, general but independent steps. The first step consists in calculating the Gram matrix (kernel matrix) using only the information of the independent variables (input); during this process, the user must define the type of kernel function that will be used in such a way as to capture the hypothesized non-linear patterns in the input data. In the second step, after the kernel matrix is built, we select the statistical machine learning algorithm to be used for training the model using the dependent variable and other available covariates, in addition to the kernel matrix from the first step. These two steps imply that we do not need to modify the statistical machine learning algorithm to accommodate a particular type of kernel function. It is important to point out that although the best known application of kernel methods is in Support Vector Machines (SVM), once the kernel matrix is ready, we can use any learning algorithm to train the model. The only important consideration to bear in mind when choosing the kernel, that it is suitable for the data at hand.

It is equally important to mention that since many machine learning methods are only able to work with linear patterns, the kernel trick allows building non-linear versions of the linear algorithms without having to modify the original machine learning algorithm. Therefore, any algorithm based on distances between objects can be formulated in terms of kernel functions, by applying the so-called “Kernel trick”. However, RKHS methods are not limited to regression; they are also very powerful for classification and data compression problems, in addition to being theoretically sound for dealing with non-linear phenomena in general. For these reasons, they have found a wide range of practical applications ranging from topics such as bioinformatics to text categorization, image analysis to web retrieval, 3D reconstruction to handwriting recognition and geostatistics to chemoinformatics (Shawe-Taylor and Cristianini 2004). The increase in popularity of kernel-based methods is due in part to the rich way in which they capture non-linear patterns in data that cannot be captured with conventional linear statistical learning methods (Gianola and van Kaam 2008; de los Campos et al. 2010; Endelman 2011). The artificial deep neural networks, random forest, and some other machine learning models do not require the kernel trick and can still work with non-linear patterns without modifying the original machine learning algorithm.

In GS, the application of RKHS methods continues to increase; for example, Long et al. (2010) found better performance of RKHS methods over linear models in the body weight of broiler chickens. Crossa et al. (2010) compared RKHS versus Bayesian Lasso and found that RKHS was better than Bayesian Lasso in the wheat data set, whereas similar performances of both methods were observed in the maize data set. Cuevas et al. (2016, 2017, 2018) also found a superior performance of RKHS methods over linear models using Gaussian kernels in maize and wheat data. Cuevas et al. (2019) found that through the use of pedigree, markers and near infrared spectroscopy (NIR) data—an inexpensive and non-destructive high-throughput phenotyping technology for predicting unobserved line performance in plant breeding trials—kernel methods (Gaussian kernel and arc-cosine kernel) outperformed linear models in terms of prediction performance. The degree of superiority of kernel methods over linear models is both data and kernel-function dependent. For this reason, we can expect significant improvements when applying the right kernel to data sets with complex non-linear patterns.

Nevertheless, other authors found minimal differences between RKHS methods and linear models, as exemplified by Tusell et al. (2013) in their study on litter size in swine; Long et al. (2010) and Morota et al. (2013) in dairy sires progeny test; and Morota et al. (2014) in phenotypes of dairy cows. These publications have empirically shown equal or better prediction ability of RKHS methods over linear models, and for this reason, the application of kernel methods in GS is expected to increase since these can be implemented in current genomic prediction software. In addition, these methods are a) very flexible; b) good for making predictions in complex settings, even though they can be difficult to interpret because of the similarity function, especially when working with purely mathematical functions lacking biological basis under their construction; and c) theoretically appealing for accommodating cryptic forms of gene action (Gianola et al. 2006; Gianola and van Kaam 2008). Likewise, these methods can be used with almost any type of information (e.g., covariates, strings, images and graphs) (de los Campos et al. 2010), and computation is performed in an *u*-dimensional space even when the original input information has more columns (*p*) than observations (*n*), thus avoiding the *p* >> *n* problem (de los Campos et al. 2010). Finally, kernel methods can also be appreciated for providing a new perspective, and while we are still far from completely understanding these methods, they are attractive for their computational efficiency, robustness and stability.

Based on the previous considerations, the main objective of this study is to provide a user-friendly way of implementing regression and classification methods based on kernels in the statistical R software. Additionally, we cover the essentials of kernel methods, as well as examples on how to handcraft an algorithm of a kernel for applications in the context of genomic selection.

## Material and methods

### Data sets and data availability

#### Wheat data set

This is a toy data set composed of 30 lines, three environments (JBL, LDH, PUS) and two continuous traits: grain yield (GRYLD) and days to heading (DTHD). This data set contains 29,157 markers coded 0, 0.5 and 1. The R file Data_Wheat_2019 RData contains the phenotypic and genotypic data and is available at https://hdl.handle.net/11529/10548532.

#### EYT data set

This wheat toy data set is composed of 40 lines, four environments (Bed5IR, EHT, Flat5IR, LHT), and four response variables: DTHD, days to maturity (DTMT), grain yield and height. In terms of genomic information, this data set contains the G_Toy_EYT genomic relationship matrix with dimension of 40 × 40. The first two traits are ordinal with three categories, the third is continuous and the last one (Height) is binary. The phenotypic and genotypic data set is available in the Data_Toy_EYT.RData (see link https://hdl.handle.net/11529/10548532). It is important to point out that all traits in the original data set are continuous and were converted to ordinal and binary data for the purpose of illustration.

### Kernel functions

A kernel function transforms information on a pair of items into a quantitative measurement representing its proximity with the restriction, and as such, the function must create a symmetric positive semi-definite (psd) matrix when applied to any subgroup of items. From a statistical standpoint, the kernel matrix can be viewed as a covariance matrix used to non-linearly transform the predictors (input) data *x*_1_,…,*x*_*p*_ ∈ *χ* into a high-dimensional feature space. For this reason, a kernel function, *K*, is defined as a “similarity” function that corresponds to an inner product in some expanded feature space that for all *x*_*i*_, *x*_*j*_ ∈ X satisfies:

$${\mathrm{K}}\left( {{\boldsymbol{x}}_i,{\boldsymbol{x}}_j} \right) = \varphi \left( {{\boldsymbol{x}}_i} \right)^{\mathrm{T}}\varphi \left( {{\boldsymbol{x}}_j} \right)$$

Where *φ*() is a mapping (transformation) function that translates the input from one space to another high-dimensional space. We can understand the feature space as the *u*- dimensions where inputs (independent variables) live. The kernel has the following properties:

(A) It is a symmetric function of its argument so that K(***x***_*i*_, ***x***_*j*_) = K(***x***_*j*_, ***x***_*i*_).
(B) To be considered valid, a kernel must fit the function K(**x**, ***x***_*j*_) (Shawe-Taylor and Cristianini 2004), where the kernel matrix **K**, also called Gram matrix, should be positive and semidefinite for all possible choices of the set (***x***_*i*_).
(C) Kernels are all those functions K(***u***,***v***) that verify Mercer’s theorem, that is, for which

$$\mathop {\int}\nolimits_{{\boldsymbol{u}},{\boldsymbol{v}}} {{\mathrm{K}}\left( {{\boldsymbol{u}},{\boldsymbol{v}}} \right)g\left( {\boldsymbol{u}} \right)g\left( {\boldsymbol{v}} \right)d{\boldsymbol{u}}d{\boldsymbol{v}}\, > \,0}$$

for all (**u**) square-integrable functions.

Below, we provide kernel functions to build some popular kernels.

#### Linear kernel

This kernel is defined as $K({\boldsymbol{x}}_{i},{\boldsymbol{x}}_{j})={\boldsymbol{x}}_{i}^{T}{\boldsymbol{x}}_{j}$ (Shawe-Taylor and Cristianini 2004). For example,

$$K\left( {{\boldsymbol{x}},{\boldsymbol{z}}} \right) = \left( {x_1,x_2} \right)\left( {\begin{array}{*{20}{c}} {z_1} \\ {z_2} \end{array}} \right) = x_1z_1 + x_2z_2 = \varphi \left( {\boldsymbol{x}} \right)^T\varphi \left( {\boldsymbol{z}} \right)$$

This kernel leaves the original representation unchanged, that is, it does not overcome the linearity limitation of linear classification and linear regression models. This means, that the mapping function, *φ*(), in this kernel is the identity function, as the input is unaltered.

#### Polynomial kernel

This kernel is defined as $K\left( {x_i,x_j} \right) = \left( {\gamma x_i^Tx_j + a} \right)^d$ where *a* is a real scalar and *d* is a positive integer, and where *γ* > 0, *a* ≥ 0, and *d* > 0 are parameters (Shawe-Taylor and Cristianini 2004). For example, when *γ* = 1, *a* = 1 and *d* = 2 we have:

$$\begin{array}{l}K\left( {x,z} \right) = \left( {\left( {x_1,x_2} \right)\left( {\begin{array}{*{20}{c}} {z_1} \\ {z_2} \end{array}} \right) + 1} \right)^2 = \left( {x_1z_1 + x_2z_2 + 1} \right)^2 \\= 1 + 2x_1z_1 + 2x_2z_2 + x_1^2z_1^2 + x_2^2z_2^2 + 2x_1z_1x_2z_2\\ = \left[ {1,\sqrt 2 x_1,\sqrt 2 x_2,x_1^2,\sqrt 2 x_1x_2,x_2^2} \right]\left[ {\begin{array}{*{20}{c}} {\begin{array}{*{20}{c}} 1 \\ {\begin{array}{*{20}{c}} {\sqrt 2 z_1} \\ {\begin{array}{*{20}{c}} {\sqrt 2 z_2} \\ {z_1^2} \end{array}} \end{array}} \end{array}} \\ {\sqrt 2 z_1z_2} \\ {z_2^2} \end{array}} \right] = \varphi \left( {\it{x}} \right)^T\varphi \left( {\it{z}} \right)\end{array}$$

The dimension of the feature space for the polynomial kernel is equal to $$\left( {\begin{array}{*{20}{c}} {n + d} \\ d \end{array}} \right)$$.

#### Sigmoidal kernel

This kernel is defined as $K\left( {x_i,x_j} \right) = tanh\left( {x_i^Tx_j + a} \right)$, where *tanh* is the hyperbolic tangent defined as: ${\mathrm{tanh}}\left( {\mathrm{z}} \right) = {\mathrm{sinh}}\left( {\mathrm{z}} \right)/{\mathrm{cosh}}\left( {\mathrm{z}} \right) = \frac{{\exp \left( z \right) - {\mathrm{exp}}\left( { - z} \right)}}{{\exp \left( z \right) + {\mathrm{exp}}\left( { - z} \right)}}$. If used with properly adjusted parameters, it can represent complex non-linear relationships. In some parameter settings, it actually becomes similar to the radial kernel. However, the sigmoid function may not be positive definite for some parameters, and therefore this does not actually represent a valid kernel (Shawe-Taylor and Cristianini 2004).

#### Gaussian kernel

This kernel, also known as the radial basis function kernel, depends on the Euclidean distance between the original attribute value vectors (i.e., the Euclidean norm of their difference) rather than on their dot product, $K\left( {{\boldsymbol{x}}_i,{\boldsymbol{x}}_j} \right) = e^{ - \gamma|| {\boldsymbol{x}}_i - {\boldsymbol{x}}||_j^2} = e^{ - \gamma \left[ {{\boldsymbol{x}}_i^T{\boldsymbol{x}}_i - 2{\boldsymbol{x}}_i^T{\boldsymbol{x}}_j + {\boldsymbol{x}}_j^T{\boldsymbol{x}}_j} \right]}$, where *γ* is a positive real scalar (Gianola and van Kaam 2008; Shawe-Taylor and Cristianini 2004). We know that the feature vector *φ* that corresponds to the Gaussian kernel is actually infinitely dimensional (Endelman 2011).

#### Exponential kernel

This kernel is defined as $K\left( {{\boldsymbol{x}}_i,{\boldsymbol{x}}_j} \right) = e^{ - \gamma|| {\boldsymbol{x}}_i - {\boldsymbol{x}}_j||}$, which is quite similar to the Gaussian kernel function (Shawe-Taylor and Cristianini 2004).

#### Arc-cosine kernel with 1 hidden layer (AK1)

For computing the AK1, first the angle between two input vectors is computed (Cuevas et al. 2019) as

$$\theta _{i,j} = \cos ^{ - 1}\left( {\frac{{{\boldsymbol{x}}_i^T{\boldsymbol{x}}_j}}{{||{{\boldsymbol{x}}_i||}\,{||{\boldsymbol{x}}_j}||}}} \right)$$

where ||***x***_*i*_|| is the norm of line *i* (Cho and Saul 2009). Finally, the AK1 is defined as

1 $${\mathrm{AK}}^1\left( {{\boldsymbol{x}}_i,\,{\boldsymbol{x}}_j} \right) = \frac{1}{\pi }||{\boldsymbol{x}}_i||\,||{\boldsymbol{x}}_j||\,J\,\left( {\theta _{i,j}} \right)$$

$$J\left( {\theta _{i,j}} \right) = \left[ {\sin \left( {\theta _{i,j}} \right) + \left( {\pi - \theta _{i,j}} \right)\cos \left( {\theta _{i,j}} \right)} \right]$$

*Arc-cosine kernel with L hidden layers (AKL)*. With more than one hidden layer (*L*), Cho and Saul (2009) proposed a recursive relationship of repeating *L* times the interior product:

2 $$AK^{\left( {L + 1} \right)}\left( {{\boldsymbol{x}}_i,\,{\boldsymbol{x}}_j} \right) = \frac{1}{\pi }\left[ {AK^{\left( L \right)}\left( {{\boldsymbol{x}}_i,\,{\boldsymbol{x}}_i} \right)AK^{\left( L \right)}\left( {{\boldsymbol{x}}_j,\,{\boldsymbol{x}}_j} \right)} \right]^{\frac{1}{2}}J\left( {\theta _{i,j}^{\left( L \right)}} \right)$$

where $\theta _{i,j}^{\left( L \right)} = cos^{ - 1}\left\{ {AK^{\left( L \right)}\left( {{\boldsymbol{x}}_i,\,{\boldsymbol{x}}_j} \right)\left[ {AK^{\left( L \right)}\left( {{\boldsymbol{x}}_i,{\boldsymbol{x}}_i} \right)AK^{\left( L \right)}\left( {{\boldsymbol{x}}_j,\,{\boldsymbol{x}}_j} \right)} \right]^{ - \frac{1}{2}}} \right\}$. *AK*^(1)^ is computed with Eq. (1) (Cuevas et al. 2019).

It should be noted that many hybrid kernels can be constructed with the previously mentioned kernels. We understand hybrid kernels as two or more simpler kernels that are combined, since complex kernels can be created by simple operations (multiplication, sum, etc.). An example of a hybrid kernel can be obtained by multiplying the polynomial kernel and the Gaussian kernel. This kernel is defined as $\left( {{\boldsymbol{x}}_i^T{\boldsymbol{x}}_j + a} \right)^de^{ - \gamma|| {\boldsymbol{x}}_i - {\boldsymbol{x}}_j||}$. However, other types of kernels can be combined in the same fashion or with other basic operations, like averaging kernels, multiplying kernels, summing kernels, etc.

The functional form of the mapping φ(**z**) does not need to be known since it is implicitly defined by the choice of kernel: *K*(**x**,**z**) = *φ*(**x**)^*T*^*φ*(**z**) or the inner product in a feature space (the feature space must therefore be a pre-Hilbert or inner product space). With a suitable choice of kernel, the data can become separable in the feature space despite being non-separable in the original input space: hence kernel substitution provides a route for obtaining non-linear algorithms from those previously restricted to handling linearly separable datasets. Thus, for example, whereas data for n-parity or the two spirals problem are non-separable by a hyperplane in an input space, they can be separated in the feature space defined by Gaussian kernels or another type of kernel such as those described above.

### Sparse kernel under a predictor with Environment + Genotype + Genotype × Environment interaction

According to Cuevas et al. (2020), a predictor that contains Environment + Genotype + Genotype × Environment interaction, under sparse kernels can be written as

3 $${\boldsymbol{y}} = \mu {\boldsymbol{1}} + {\boldsymbol{Z}}_{\boldsymbol{E}}\beta _{\boldsymbol{E}} + {\boldsymbol{P}}^{u_1}{\boldsymbol{f}} + {\boldsymbol{P}}^{u_2}{\boldsymbol{l}} + {\boldsymbol{\varepsilon}}$$

where *μ* is the overall mean, and **1** is the vector of ones. The fixed effects of environments are modeled with the incidence matrix of environments ***Z***_*E*_. The parameters to be estimated are the intercept for each environment (***β***_*E*_). **P**^*u*1^ = **Z**_*u*1_**P** of order n^*^ × *m*, with n^*^ = *n*_1_ + *n*_2_ + ··· + *n*_*I*_, **Z***u*_1_ is the incidence matrix that relates the genotypes to the phenotypic observations and where ***P*** is computed as **P** = **K**_*L*,*m*_*US*^−1/2^ and ***f*** is a vector of *m* × 1; **P**^*u*2^ = P^*u*1^:***Z***_*E*_ of order n^*^ × *mI* and vector ***l*** is of order *mI* × *L*, and the notation ***P***^*u*1^:***Z***_*E*_ denotes the interaction term between the design matrix ***P***^*u*1^ and ***Z***_*E*_. The third term in equation (6) approximates the term ${\boldsymbol{u}}_1\sim {\boldsymbol{N}}( {0,\sigma _{u_1}^2{\boldsymbol{K}}_1})$, with ${\boldsymbol{K}}_1 = {\boldsymbol{Z}}_{u1}{\boldsymbol{KZ}}_{u1}^\prime$, while the fourth term approximates the term ${\boldsymbol{u}}_2\sim {\boldsymbol{N}}( {0,\sigma _{u_2}^2{\boldsymbol{K}}_2})$, where ${\boldsymbol{K}}_2 = \left( {{\boldsymbol{Z}}_{u1}{\boldsymbol{KZ}}_{u1}^\prime } \right) \circ \left( {{\boldsymbol{Z}}_E{\boldsymbol{Z}}_E^\prime } \right)$, where ° is the Hadamard product.

Next we provide all the details on the computation of the **P**^*u*1^ and **P**^*u*2^ design matrices that result from using the approximate kernel:

**Step 1:** We assume that we have a matrix **X** that contains the markers of each line without replication, i.e., each row corresponds to a different line. We assume that this matrix contains *L* lines (rows) and *p* markers (columns). It is important to point out that this matrix is standardized by columns.

**Step 2:** We randomly select *m* lines out of the *L* from the training set **X** only one time.

**Step 3:** Next we construct kernel matrices ***K***_*m*,*m*_ and ***K***_*L,m*_, from the matrix of markers ***X***_*m,p*_ and ***X***_*L,p*_, respectively. For example, for the linear kernel, kernel matrices ***K***_*m,m*_ and ***K***_*L,m*_ are computed as: ${\boldsymbol{K}}_{m,m} = \frac{{{\boldsymbol{X}}_{m,p}{\boldsymbol{X}}_{m,p}^\prime }}{p}$, ${\boldsymbol{K}}_{L,m} = \frac{{{\boldsymbol{X}}_{L,p}{\boldsymbol{X}}_{m,p}^\prime }}{p}$. ***K***_*m,m*_ and ***K***_*L,m*_ are smaller than the original kernels of order *L* × *L* and thus, we are able to build the sparse kernel.

**Step 4:** Compute the eigen value decomposition of ***K***_*m,m*_ = ***USU***^*T*^, where ***U*** is the matrix of eigenvectors and **S** is a diagonal matrix of eigenvalues.

**Step 5:** Compute matrix ***P*** = ***K***_*L,m*_*US*^−1/2^. ***P*** is of order *L* × *m*.

**Step 6:** Compute matrix ***P***^*u*1^ = **Z**_*u*1_***P*** = Z_*u*1_***K***_*L,m*_***US***^**−**1/2^, where ***Z***_*u*1_ is the design matrix of lines of order n^*^ **×** *L* and ***P***^*u*1^ of order n^*^ × *m*.

**Step 7:** Compute matrix ***P***^*u*2^ = ***P***^*u*1^:***Z***_*E*_, where: denotes the interaction between the design matrix ***P***^*u*1^ and ***Z***_*E*_. ***Z***_*E*_ is the design matrix of environments of order n^*^ × *I* and ***P***^*u*2^ is of order n^*^ × *mI*.

**Step 8:** Fit the model under a Ridge regression framework (like BGLR or glmnet) and make genomic-enabled predictions for future data.

The approximate kernel method (explained above) with a predictor that contains Environment + Genotypes + Genotypes × Environment requires that some lines were studied in some environments, but not in all. This approximate kernel method gains efficiency when the number of environments is low and the number of lines is high. It is also important to point out that the proposed sparse method does not reduce the original number of rows existing on the data sets. Since this does not change, the reduction is in the number of input (independent variables) with which the model should be trained. For example, assume that we have three environments (*I* = 3) and each was evaluated the same. If *L* = 40 lines and we have access to *p* = 10,000 markers for each line, and then build a genomic relationship matrix of 40 × 40 with marker information, this means that the dimension of the independent variables will be equal to 3 + 40 + 40 × 3 = 163, taking into account Environment + Genotypes + Genotypes × Environment interaction in the predictor. However, under a sparse version of the linear kernel with *m* = 10, the required number of independent variables for training this model would be equal to 3 + 10 + 10 × 3 = 43, that is, a reduction of $\left( {163 - 43} \right) \times \frac{{100}}{{163}} = 73.61\%$ in the number of inputs. This is because the input corresponding to the lines is reduced from 40 columns to ten.

### Evaluation of prediction performance

To evaluate the prediction performance of kernel methods with the illustrative examples provided in the results section, we used a ten-fold cross-validation strategy that consisted in the following steps: (1) data was randomly divided into ten mutually exclusive subsets, (2) we performed the prediction for each fold using 9 folds as training each time (3) we computed the metrics for each fold in the testing sets and 4) we averaged the ten metrics that were reported as prediction performance of each model (Theodoridis 2020). The metrics used were the mean square error (MSE) for continuous response variables, the proportion of cases correctly classified (PCCC) and the Kappa coefficient for ordinal response variables. The ***Kappa coefficient*** or Cohen’s Kappa, is defined as: $\kappa = \frac{{P_0 - P_e}}{{1 - P_e}}$; where *P*_0_ is the agreement between observed and predicted values and is computed by the PCCC described above for two classes; *P*_*e*_ is the probability of agreement calculated as $P_e = \frac{{tp\, + \,fn}}{n} \times \frac{{tp\, + \,fp}}{n} + \frac{{fp\, + \,tn}}{n} \times \frac{{fn\, + \,tn}}{n}$, where *fp* is the number of false positives, and *fn* is the number of false negatives. This statistic can take on values between −1 and 1,where a value of 0 means there is no agreement between the observed and predicted classes and a value of 1 indicates perfect agreement between the model prediction and the observed classes (González-Camacho et al. 2018). Additionally, we calculated the corresponding standard error (SE) for each metric. It is important to point out that the ten-fold cross-validation strategy was implemented with only one replication. To implant the Bayesian kernel methods, we used the BGLR library of Pérez-Rodríguez and de los Campos (2014) in the R statistical software (R Core Team 2020).

## Results

The results are given in four sections. In the first section we show how to compute the kernels explained in Material and Methods, while in the second section, we provide examples for implementing kernel methods for continuous response variables under single-environment, multi-environment and multi-trait analysis, respectively. The third section highlights examples of multi-environment analysis for ordinal response variables, and the fourth section provides examples for building sparse kernels and for implementing them under a multi-environment framework for continuous response variables.

### Kernel matrix construction

To illustrate kernel construction, we only use 6 lines and 5 markers of the data set Data_Wheat_2019. RData. These 6 lines and 5 markers are given below.

> XF

**Table:** 

	M1	M2	M3	M4	M5
GID304660	−0.997	−0.997	−0.997	−0.997	−0.997
GID6175067	0.427	0.427	0.427	0.427	0.427
GID6332122	−0.421	−2.421	−2.421	−2.421	−2.421
GID6341870	0.427	0.427	0.427	0.427	0.427
GID6931427	0.427	0.427	0.427	0.427	0.427
GID7460318	0.427	0.427	0.427	0.427	0.427

To compute the linear kernel using the kernel.construction(), the function given in Appendix A, we need to use the following lines of code:

> source(“Kernel_construction.R”)> K.Linear=Kernel_computation(X=XF,name=“Linear”, degree=NULL, nL=NULL)> round(K.Linear,3)

**Table:** 

	GID304660	GID6175067	GID6332122	GID6341870	GID6931427	GID7460318
GID304660	0.994	−0.426	2.414	−0.426	−0.426	−0.426
GID6175067	−0.426	0.182	−1.034	0.182	0.182	0.182
GID6332122	2.414	−1.034	5.861	−1.034	−1.034	−1.034
GID6341870	−0.426	0.182	−1.034	0.182	0.182	0.182
GID6931427	−0.426	0.182	−1.034	0.182	0.182	0.182
GID7460318	−0.426	0.182	−1.034	0.182	0.182	0.182

The newly computed information is the kernel matrix under a linear kernel. In a similar fashion, to compute the polynomial, the sigmoid, the Gaussian, exponential, the AK1 and AKL kernels, we need to specify the corresponding kernel names in Name=“ ” in the Kernel_computation() function. However, for the polynomial kernel, it is necessary to specify the degree and for the AKL, the number of hidden layers (deep kernel) in the nL parameter. The complete code for computing these kernels is given in Appendix B.

### Kernel implementation for continuous response variables

In this section we provide illustrative examples for single-environment analysis, multi-environment analysis and multi-trait analysis.

#### Single environment data

The base BGLR code for implementing Bayesian kernel single-environment analysis.

*K_Lines=Kernel_computation(X=XF,name=Gaussian, degree=NULL,nL=NULL)*

K_expanded=Z_L%*%K_Lines%*%t(Z_L)

*ETA=list(list(model=‘RKHS’, K=K_expanded))*

*y_NA = y*

*y_NA[Pos_tst] = NA*

*A = BGLR(y=y_NA, ETA=ETA, nIter = 1e4, burnIn = 1e3, verbose = FALSE)*

*yp_ts = A$yHat [Pos_tst]*

In kernel_computation() it is necessary to specify the required kernel method to be used, as illustrated in the previous section. Then, this kernel is expanded to take into account the replications available in the data set for each line using the design matrix of lines *Z_L*. All details for building this design matrix (*Z_L*) are given in Appendix C. Then, a list called ETA is created in which the expanded kernel is provided in K, and the model is specified as RKHS. Then in the original response variable, those observations that belong to the testing set in each partition are set to NA, to avoid using them in the training process. In the BGLR() function these NA are provided in y, such that the response variable with NA belongs to the testing set, while in ETA, the list that contains the expanded kernel is provided and the model RKHS method is specified. Here, we also specify the number of iterations (nIter), burning (burnIn) and a logical value (TRUE and FALSE) for printing (or not) the training process. Finally, the predictions are extracted with *A$yHat* and only the predictions for the testing set are saved in *yp_ts*.

For the training process we only used one environment (JBL) of the Data_Wheat_2019.RData data set. The number of lines in this environment was 30 and some had more than one replication. A ten-fold cross-validation was implemented using nine for training and one for testing, and the average of the ten testing folds was reported as the prediction performance. The results are given in Table 1, and the R code for its implementation is provided in Appendix C. Table 1 indicates that the best prediction performance was observed under the AKL kernel that used five hidden layers (kernel deep), while the worst kernel was the sigmoid kernel.

**Table 1:** Single-environment prediction performance of 7 kernels under a ten-fold cross-validation with data Data_Wheat_2019.RData.

Kernel	MSE	SE_MSE	Cor	SE_Cor	Time
Linear	5.279	0.497	0.642	0.048	10.140
Polynomial	4.974	0.587	0.658	0.054	10.510
**Sigmoid**	**5.594**	**0.492**	**0.625**	**0.049**	**9.370**
Gaussian	4.881	0.540	0.668	0.047	12.440
AK1	5.149	0.522	0.651	0.047	10.600
**AKL**	**4.754**	**0.515**	**0.678**	**0.047**	9.970
Exponential	4.980	0.521	0.663	0.048	9.780

MSE denotes the mean square error of prediction (SE_MSE id the standard error of MSE) and Cor is used for the average Pearson’s correlation (SE_Cor is the standard error of Cor). Time indicates the implementation time in seconds.

#### Multi-environment data

The BGLR code for implementing Bayesian kernel methods with an extended predictor (main effects of environment and genotypes and the G × E term) is provided as:

*Z_E=model.matrix(~0+Env,data=Pheno)*

dim(Z_E), K.E=Z_E%*%t(Z_E)

*K_Lines=Kernel_computation(X=XF,name=kernel_name[i], degree=2,nL=5)*

K_expanded=Z_L%*%K_Lines%*%t(Z_L)

K_GE=K_expanded*K.E

*ETA=list(Env=list(model=‘FIXED’,X=Z_E),Lines=list(model=‘RKHS’,K=K_expanded),GE=list(model=‘RKHS’,K=K_GE))*

*y_NA = y*

*y_NA[Pos_tst] = NA*

*A = BGLR(y=y_NA,ETA=ETA,nIter = 1e4,burnIn = 1e3,verbose = FALSE)*

*yp_ts = A$yHat [Pos_tst]*

The only difference in the code with the single-environment analysis is that now in the ETA (predictor), the effects of environment and G×E are specified in addition to the genotype effects, that is, three terms are included in the predictor. However, the kernel for lines expanded, takes into account the incidence matrix of lines, *Z_L*. Also, a kernel was created for the G×E that was obtained as the Hadamard product between the expanded kernel of genotypes and environments. However, the main effects of environments are considered fixed effects, using only the incidence matrix of environments, *Z_E*. The whole code of this example is given in Appendix D.

Table 2 shows that in terms of MSE, the best prediction performance was obtained with the AKL kernel with kernel deep = 5. However, in terms of Pearson’s correlation, the best prediction performance was obtained in the polynomial, Gaussian and AKL kernels. It is important to point out that the worst implementation time was with the AKL (76.29 s) kernel since for its implementation, we assumed that the AK1 was already estimated, and its computation required an iterative process. The R code for reproducing the results in Table 2 is given in Appendix D.

**Table 2:** Multi-environment prediction performance of seven kernels under a ten-fold cross-validation with data Data_Wheat_2019.RData.

Kernel	MSE	SE_MSE	Cor	SE_Cor	Time
Linear	2.916	0.240	0.988	0.001	33.560
Polynomial	2.753	0.235	0.989	0.001	36.400
Sigmoid	3.148	0.262	0.987	0.001	35.090
Gaussian	2.723	0.219	0.989	0.001	40.450
AK1	2.819	0.230	0.988	0.001	40.730
AKL	2.670	0.225	0.989	0.001	76.29
Exponential	2.919	0.232	0.988	0.001	31.390

MSE denotes the mean square error of prediction (SE_MSE id the standard error of MSE) and Cor is used for the average Pearson’s correlation (SE_Cor is the standard error of Cor). Time indicates the implementation time in seconds.

#### Multi-trait data

The multi-trait prediction model with the same predictor (Environment + Genotype + G × E) that was previously used for the multi-environment example, is similar to that used for multi-environment analysis since the specification of the predictor (ETA) is exactly the same. However, the function used for implementing the multi-trait genome prediction models is different [now *Multitrait()]*; other details of the code are given below. The complete code is given in Appendix E and the output is given in Table 3.

**Table 3:** Multi-trait prediction performance of seven kernels under a ten-fold cross-validation with data Data_Wheat_2019.RData.

Kernel	Trait	MSE	SE_MSE	Cor	SE_Cor
Linear	DTHD	2.686	0.355	0.848	0.027
Linear	GRYLD	0.317	0.038	0.541	0.064
Polynomial	DTHD	2.626	0.380	0.850	0.029
Polynomial	GRYLD	0.314	0.039	0.544	0.062
Sigmoid	DTHD	2.877	0.376	0.837	0.029
Sigmoid	GRYLD	0.326	0.038	0.526	0.065
Gaussian	DTHD	2.533	0.349	0.855	0.028
**Gaussian**	**GRYLD**	**0.313**	**0.037**	**0.549**	**0.061**
AK1	DTHD	2.571	0.345	0.854	0.027
**AK1**	**GRYLD**	**0.313**	**0.036**	**0.550**	**0.061**
**AKL**	**DTHD**	**2.440**	**0.344**	**0.859**	**0.028**
AKL	GRYLD	0.315	0.036	0.539	0.060
Exponential	DTHD	2.551	0.342	0.852	0.028
Exponential	GRYLD	0.345	0.041	0.480	0.068

MSE denotes the mean square error of prediction (MSE) and its standard error (SE_MSE), Cor is used for the average Pearson’s correlation (Cor) and its standard error (SE_Cor).

*K_Lines=Kernel_computation(X=XF,name=kernel_name[i], degree=2,nL=5)*

K_expanded=Z_L%*%K_Lines%*%t(Z_L)

K_GE=K_expanded*K.E

*ETA=list(Env=list(model=‘FIXED’, X=Z_E),Lines=list(model=‘RKHS’, K=K_expanded),GE=list(model=‘RKHS’, K=K_GE))*

*Pos_tst =PT[,k]*

*y_NA = data.matrix(y)*

*y_NA[Pos_tst,] = NA*

*A1= Multitrait(y = y_NA, ETA=ETA, resCov = list(type =“UN”, S0=diag(2),df0= 5)*,

*nIter =10000, burnIn = 1000)*

*Metrics= PC_MM_f(y[Pos_tst,],A1$ETAHat[Pos_tst,],Env=Pheno$Env[Pos_tst])*

The building process of the design matrices and kernels was the same as the previous example. However, now in place of using the BGLR() function, which is useful for uni-trait analysis, we used the Multitrait() function, appropriate for fitting Bayesian multi-trait models in the BGLR package. In addition to the ETA and the response variable for the Multitrait() function, we must also provide the type of covariance structure, the degrees of freedom (df0) and the S0, which is usually a diagonal matrix of a dimension equal to the number of traits being used. Appendix E provides the whole code for this example.

Table 3 shows that the best prediction performance for trait DTHD was observed under both metrics (MSE and Cor) and on the Arc-cosine kernel with kernel deep equal to 5 (AK_L), while under trait GRYLD, the best prediction performance was shared by two kernels [Gaussian and Arc-cosine with only one hidden layer (kernel deep)].

### Kernel implementation for ordinal response variables

While the data used for illustrating the implementation of Bayesian kernel methods with an ordinal response variable are the same as the previously used data, the discretizing the response variable was adjusted according to the first (level 1), second (level 2) and third quantile (level 3) in each environment. The predictor was the same as the one used for the continuous response under multi-environment analysis (Environment + Genotypes + G × E). For this reason, all codes are almost the same as the ones given above for the multi-environment example with a continuous response variable, except for the following lines of code:

*A=BGLR(y=y_NA, ETA=ETA, response_type=“ordinal”, nIter = 1e4,burnIn = 1e3, verbose = FALSE)*

*Probs = A$probs[Pos_tst,]*

*yp_ts = apply(Probs,1,which.max)*

The different key components are: (a) response_type=“ordinal” that makes BGLR using the machinery of Bayesian probit regression for ordinal response variables; (b) Probs = A$probs[Pos_tst,], which extracts the probabilities of each level of the response variable for all the individuals in the testing set; and (c) yp_ts = apply(Probs,1,which.max) that transforms the probabilities of each individual to levels 1, 2 and 3 that are the possible levels of the original response variable in this situation. The complete code for reproducing results of Table 4, for Bayesian kernel regression with ordinal response variables is given in Appendix F.

**Table 4:** Multi-environment prediction performance of seven kernels with a ordinal response variable under a ten-fold cross-validation with data Data_Wheat_2019.RData.

Kernel	PCCC	SE_PCCC	Kappa	SE_Kappa	Time
Linear	0.671	0.025	0.454	0.044	62.72
Polynomial	0.680	0.026	0.468	0.049	69.98
Sigmoid	0.664	0.025	0.443	0.045	71.25
**Gaussian**	**0.689**	**0.028**	**0.483**	**0.054**	**72.1**
AK1	0.664	0.027	0.441	0.050	79.83
AKL	0.673	0.025	0.450	0.048	65.6
Exponential	0.596	0.035	0.281	0.075	72.76

PCCC denotes the proportion of cases correctly classified (SE_PCCC is the standard error of PCCC) and Kappa is average Kappa’s coefficient (SE_Kappa is the standard error of Kappa). Time indicates the implementation time in seconds.

Under an ordinal response variable, there are also differences in prediction performance between the seven implemented kernels (Table 4). Under this method, the Gaussian kernel produced the best prediction performance in terms of PCCC and Kappa coefficient.

### Implementation for sparse kernel methods

First, we will illustrate how to compute the ***P*** matrix, which is needed to compute the ***P***^*u*1^ and ***P***^*u*2^ design matrices needed in equation (6) to implement approximate kernel methods. To compute ***P*** we will use the code given in Appendix G. Appendix H contains some examples for computing the design matrix, ***P***, under seven types of kernels by using the Sparse_Kernel_Construction.R code provided in Appendix G. Next, we illustrate how to compute the ***P***, under the Gaussian kernel. Under this method, the XF matrix was used with only ten rows and all the markers. The number of columns (sample size) for ***P*** was specified as *m* = 3, while degree and nL were specified as NULL since the Gaussian kernel does not require these hyper-parameters. For this reason we can see in the output that the resulting ***P*** design matrix, whose original size was (10 × 10), was reduced to 10 × 3, showing that under a Bayesian Ridge regression model it only three beta coefficients are needed instead of ten.

source(“Sparse_Kernel_Construction_Appendix_G.R”)

> K.Gaussian1=Sparse_Kernel_Construction(m=3, X=XF, Name=“Gaussian”, degree=NULL,nL=NULL)

> round(K.Gaussian1,3)

**Table:** 

	[,1]	[,2]	[,3]
[1,]	−0.597	−0.780	−0.186
[2,]	−0.671	0.495	−0.552
[3,]	−0.129	−0.004	0.009
[4,]	−0.261	−0.016	0.076
[5,]	−0.295	−0.047	−0.018
[6,]	−0.133	0.023	0.001
[7,]	−0.206	−0.054	0.031
[8,]	−0.215	−0.056	−0.001
[9,]	−0.710	0.188	0.678
[10,]	−0.134	−0.028	−0.004

Note that If we use an *m* value of 5, in the Sparse_Kernel_Construction() function, the resulting ***P*** matrix will be of the order 10 × 5, which will reduce the number of beta coefficients that need to be estimated by 50%.

It is important to point out that when the value of *m* is smaller, the reduction in necessary parameter estimates is greater, which means a greater gain in computational resources. Therefore, we suggest choosing the value of *m* by cross-validation in such a way as to guarantee a significant reduction in time of execution of the model without any significant loss in the prediction accuracy. This means that a grid with different values of *m* should be evaluated and the optimal one obtained by cross validation should be one that is small enough to not affect the prediction performance with regard to using the whole information. Ten-fold cross validation can be used but if a higher rate of accuracy is required, this should be repeated s times. Appendix H illustrates how to compute the value of ***P*** for other types of kernels, but in general, only the name of the required kernel in the Sparse_Kernel_Construction() function must be specified, in addition to the *m* value. The key codes for building the ***P***, ***P***^*u*1^, ***P***^*u*2^ matrices and for implementing in BGLR the Bayesian sparse kernel methods are given as follows:

P_Lines=Sparse_Kernel_Construction(m=mvalues[s], X=XF,name=kernel_name[i], degree=2, nL=5)

Var0=apply(P_Lines, 2, sd)

pos_varNo0=which(Var0>0)

P_Lines1=P_Lines[,pos_varNo0]

P_expanded=Z_L%*%P_Lines1

P_GE=model.matrix(~0+P_expanded:Env,data=Pheno)

ETA=list(Env=list(model=‘FIXED’, X=Z_E),Lines=list(model=‘BRR’, X=P_expanded),GE=list(model=‘BRR’, X=P_GE))

Pos_tst =PT[,k]

y_NA = y

y_NA[Pos_tst] = NA

A = BGLR(y=y_NA,ETA=ETA, nIter = 1e4, burnIn = 1e3, verbose = FALSE)

yp_ts = A$yHat

If we want to implement other members of the Bayesian alphabet in the model (in the ETA), we only need to replace BRR with BayesA, BayesB, BayesC or BL for Bayesian Lasso.

Appendix I gives the complete R code for implementing sparse Bayesian regression that reproduces the results in Table 5. For the example, we used the data set Data_Toy_EYT.RData, which contains four environments, four traits (DTHD, DTMT, GY and Height) and 40 observations in each environment. Traits DTHD, DTMT and Height are ordinal and GY is continuous. We also provided a genomic relationship matrix (GRM) of dimension 40 × 40, and the square root matrix of the GRM was used as input for building the sparse kernels. For the illustration, we only used the continuous trait (GY) as the response variable. The approximate kernels were built using the 40 lines (square root matrix of the GRM) from which the training set was built with *m* = 10, 15, 20, 35 and 40 lines. We implemented seven (linear, polynomial, sigmoid, Gaussian, AK1, AKL and Exponential) sparse kernels.

**Table 5:** Prediction performance in terms of mean square error (MSE) and its standard error (SE_MSE), as well as Pearson’s correlation and its standard error (SE_Cor) under seven approximate Gaussian kernel methods with the method proposed by Cuevas et al. (2020).

m	Kernel	MSE	SE_MSE	Cor	SE_Cor	Time
10	Linear	0.348	0.045	0.908	0.026	13.79
10	Polynomial	0.349	0.044	0.911	0.024	15.98
10	**Sigmoid**	**0.307**	**0.034**	**0.922**	**0.021**	**16.19**
10	**Gaussian**	**0.300**	**0.029**	**0.926**	**0.018**	**18.52**
10	AK1	0.344	0.028	0.915	0.021	16.53
10	AKL	0.314	0.035	0.921	0.020	15.2
10	Exponential	0.336	0.027	0.918	0.018	15.56
15	Linear	0.318	0.036	0.917	0.022	17.72
15	Polynomial	0.344	0.031	0.909	0.021	18.92
15	Sigmoid	0.338	0.035	0.913	0.023	19.13
15	Gaussian	0.308	0.035	0.922	0.021	20.44
15	AK1	0.332	0.034	0.915	0.022	20.14
15	AKL	0.336	0.023	0.919	0.018	21.16
15	Exponential	0.353	0.033	0.912	0.021	19.58
20	Linear	0.316	0.034	0.918	0.021	18.31
20	Polynomial	0.346	0.036	0.910	0.025	18.33
20	**Sigmoid**	**0.281**	**0.034**	**0.926**	**0.021**	**19.73**
20	**Gaussian**	**0.306**	**0.034**	**0.924**	**0.021**	**15.72**
20	AK1	0.345	0.025	0.916	0.020	15.35
20	AKL	0.323	0.028	0.918	0.021	15.25
20	Exponential	0.346	0.035	0.914	0.023	16.64
35	Linear	0.303	0.026	0.924	0.018	19.39
35	Polynomial	0.316	0.027	0.921	0.020	20.35
35	Sigmoid	0.292	0.025	0.926	0.019	22.24
35	Gaussian	0.327	0.027	0.920	0.020	22.91
35	AK1	0.333	0.025	0.918	0.020	23.5
35	AKL	0.324	0.028	0.921	0.019	21.61
35	Exponential	0.330	0.024	0.920	0.020	22.89
40	Linear	0.307	0.026	0.923	0.019	24.03
40	Polynomial	0.326	0.026	0.919	0.020	24.14
40	Sigmoid	0.306	0.026	0.923	0.019	22.25
40	Gaussian	0.322	0.025	0.921	0.019	21.29
40	AK1	0.334	0.024	0.919	0.019	25.25
40	AKL	0.333	0.023	0.920	0.019	23.69
40	Exponential	0.335	0.023	0.919	0.019	20.63

Ten-fold cross-validation was implemented and the prediction performance was only reported for the testing set. Five values of training size, m, were implemented: 5, 10, 20, 35 and 40 (all data). Time reported: the implementation time in seconds. The data set used for this example was Data_Toy_EYT with trait GY as the response variable.

Table 5 shows that there are differences in the prediction performance using different kernels. However, the approximate kernel, even with *m* = 10, many times outperformed the prediction performance of the exact kernel (*m* = 40), which implies that when the lines are highly correlated even with a small sample size, *m*, approximating the kernel is enough to get decent prediction performance. However, the time gained by using a sample size, *m*, is significantly lower than using the total number of lines, and this gain in implementation time is more relevant when the number of lines is very large, as was stated by Cuevas et al. (2020). In general, Table 5 indicates that the best prediction performance in terms of MSE and average Pearson’s correlation was observed with *m* = 20. With this data set, only 25% of the lines (*m* = 10 lines) is enough to approximate the exact kernel (with 40 lines) quite well; however, the value of *m* is data dependent. It is important to point out that the code provided in Appendix I can easily be adapted for ordinal variables since the only requirement is to provide an ordinal response variable and specify *response_type=“ordinal”* inside the BGLR() function. For single-environment analysis, the only requirement is to provide the term related to genotypes in the predictor (ETA). Implementation of the Sparse kernel version under a multi-trait framework is similar to the uni-trait example since the process for constructing the design matrices ***P***, ***P***^*u*1^ and ***P***^*u*2^ is the same.

## Discussion

The goal of this study was to provide researchers with a user-friendly guide for implementing Bayesian kernel methods for continuous, binary and ordinal response variables in the context of genomic prediction. Seven different types of kernels were illustrated with real toy data sets since there is empirical evidence showing that not all kernels perform well in specific data sets. However, there is also empirical evidence that kernel methods more frequently outperform conventional linear machine learning methods when data sets contain complex non-linear patterns that cannot be captured by linear machine learning models (Ober et al. 2011).

The most popular kernel classification/regression method is support vector machine (SVM) since it has a good generalization performance in many real-life data sets; in addition, there are few free parameters to adjust and the architecture of the learning machine does not need to be found by experimentation. Moreover, all the virtues of kernel methods are not only valid for SVM, but also for Bayesian kernel methods, as was illustrated by the examples provided in the results section. These facts have been supported by many of the publications on Bayesian kernel methods by Cuevas et al. (2016, 2017, 2018, 2019 and 2020). In this paper, we illustrated the implementation of Bayesian kernel methods by focusing mainly on some well-known kernel methods (linear, polynomial, sigmoid, Gaussian, exponential and arc-cosine 1 and arc-cosine L) that frequently outperform the linear kernel.

There are different approaches for capturing non-linear patterns from input data, such as the so-called reproducing kernel Hilbert spaces (RKHS) was proposed under a very friendly framework. RKHS maps the input variables into a new space, such that the original non-linear pattern is transformed into a linear one. The power and beauty of RKHS methods is that their rich structure allows us to perform inner product operations in a very efficient way, with complexity unrelated to the dimensionality of the respective RKHS (Shawe-Taylor and Cristianini 2004). Additionally, RKHS methods are able to build non-linear versions of any linear algorithms by replacing their independent variables (predictors) with a kernel function, producing machines with greater power and better prediction performance. Although the dimension of such spaces can even be infinite (Theodoridis 2020), kernels are interpreted as scalar products in high-dimensional spaces.

Most of the seven kernels studied in this guide have analytical solutions (linear, polynomial, sigmoid, Gaussian, AKL, and exponential); however, the AKL requires an iterative solution. For this reason, the computation of the AKL kernel is most demanding, and the larger the kernel deep (number of hidden layers), the longer its computation time. Although only seven kernel methods were computed, they possess great versatility and composite kernels can be built; some can be computed in closed form, while others require an iterative process. Our illustrative examples show that kernel methods can also be implemented under the conventional Bayesian regression framework, and the current software for genome-enabled prediction can be used for implementing kernel Bayesian methods. This is possible due to a two-step process used to implement Bayesian kernel methods: (a) the kernel is computed in the first step and (b) in the second step, this kernel is incorporated into conventional Bayesian methods for genomic prediction.

The goal of this paper is to facilitate the implementation of kernel methods, since there is sufficient empirical evidence showing that they are remarkably flexible and exploit complexity to improve prediction accuracy. Their flexibility is attributed in part to the fact that they can be implemented using statistical and machine linear algorithms. In this guide, the Bayesian linear regression algorithms were used to implement kernel methods; however, conventional linear mixed models can also be used for implementing kernel methods since a two-step process is required for their implementation (Endelman 2011). Kernel methods can be implemented in popular machine learning algorithms like support vector machine, random forest, artificial neural networks and deep learning methods, among others. It is important to point out that kernel methods can be seen as artificial neural network methods since they work with transformed inputs, which is equivalent to implementing one hidden layer, with the kernels equivalent to the activation function in the hidden layer. Since it requires transforming the original input more than once (kernel deep), the AKL kernel imitates the deep learning method with regard to the number of hidden layers. However, AKL kernel, as pointed out by one reviewer, does not contain learnable parameters for hidden layers as deep neural networks where gradients are propagated during optimization. The AKL kernel is only a recursive application of a deterministic similarity function.

In the illustrative examples of approximate kernels provided, we observed that by using the sparse kernels, we can obtain a relevant reduction in time without a significant reduction in prediction accuracy. According to Cuevas et al. (2020), this method is preferred for very large data sets where more gain is obtained in the implementation time. Sparse kernel methods provide promising tools for large-scale and high-dimensional genomic data processing. For this reason, while these kernels, based on data compression ideas, are very promising for very large data sets, software and more research are still needed to simultaneously develop new methods and improve existing ones.

Although kernel methods have advantages and exploit complexity to improve prediction accuracy, they are unhelpful at increasing the understanding of the complexity, and as such it is important to avoid false expectations about these methods. Nevertheless, although this subject is still very much under development, these methods are an important tool for machine learning and applications since they frequently increase prediction accuracy when the patterns in the input data are non-linear. Kernel methods also promote further improvement in the scalability of conventional machine learning methods because of their versatility to work with heterogeneous inputs since kernel methods guarantee existence and uniqueness, just like least square methods.

Finally, the given examples are representative of many types of analyses required for plant breeding for genomic-enabled prediction: single-environment analysis, multi-environment analysis and multi-trait analysis with continuous, binary and ordinal response variables. For this reason, we believe that this guide will help scientists implement Bayesian kernel methods using their own data with slight modifications to the code (as provided). Finally, since the implementation of the kernels methods was done in a two-step process, other algorithms like conventional mixed models, albeit with small modifications, can be used for their implementation.

## Conclusions

This paper provides a guide for implementing kernel methods for genomic prediction. The R code is provided for building the kernels (linear, polynomial, sigmoid, Gaussian, AK1, AKL and exponential) and for implementing exact and approximate kernel Bayesian methods under a single- environment, multi-environment and multi-trait framework. We provided examples for univariate continuous variables, as well as for binary and ordinal outcomes, whereas for multivariate variables, an example for only continuous response variables was illustrated. Here, kernel methods were implemented using a two-step process where the kernel was computed in the first step, which was subsequently used to implement Bayesian methods for genomic prediction in the second. The approximate kernel methods were implemented only for Bayesian Ridge regression (BRR), but their implementation is straightforward for BayesA, BayesB, BayesC and Bayesian Lasso under a univariate framework. Additionally, the prediction performance and implementation time of the approximate kernel were compared to exact kernel (that use all the information) methods, and we observed that, in terms of prediction performance, approximate kernels methods were very competitive with exact kernel methods, but there was a significant reduction in computational resources since the implementation time of the approximate kernels is considerably low.

## Data Availability

The two data sets, R file Data_Wheat_2019.RData, and Data_Toy_EYT.RData can be found at the following link https://hdl.handle.net/11529/10548532.

## Appendix A. Code for computing the linear, polynomial, sigmoid, Gaussian, AK1, AKL, and exponential kernels

Kernel_computation=function(X,name, degree, nL){

p=ncol(X)

x1=X

x2=X

d=degree

############Polynomial kernel##################

K.Polynomial=function(x1, x2=x1, gamma=1, b=0, d=3)

{ (gamma*(as.matrix(x1)%*%t(x2))+b)^d}

############Sigmoid kernel####################

K.Sigmoid=function(x1,x2=x1, gamma=1, b=0)

{ tanh(gamma*(as.matrix(x1)%*%t(x2))+b) }

############Gaussian kernel##################

l2norm=function(x){sqrt(sum(x^2))}

K.Gaussian=function(x1,x2=x1, gamma=1){

exp(-gamma*outer(1:nrow(x1<- as.matrix(x1)), 1:ncol(x2<- t(x2)),

Vectorize(function(i, j) l2norm(x1[i,]-x2[,j])^2)))}

##########Arc-cosine kernel with 1 hidden layer

K.AK1_Final<-function(x1,x2){

n1<-nrow(x1)

n2<-nrow(x2)

x1tx2<-x1%*%t(x2)

norm1<-sqrt(apply(x1,1,function(x) crossprod(x)))

norm2<-sqrt(apply(x2,1,function(x) crossprod(x)))

costheta = diag(1/norm1)%*%x1tx2%*%diag(1/norm2)

costheta[which(abs(costheta)>1,arr.ind = TRUE)] = 1

theta<-acos(costheta)

normx1×2<-norm1%*%t(norm2)

J = (sin(theta)+(pi-theta)*cos(theta))

AK1 = 1/pi*normx1×2*J

AK1<-AK1/median(AK1)

colnames(AK1)<-rownames(x2)

rownames(AK1)<-rownames(x1)

return(AK1)

}

####Kernel Arc-Cosine with deep=L#########

AK_L_Final<-function(AK1,nL){

n1<-nrow(AK1)

n2<-ncol(AK1)

AKl1 = AK1

for (l in 1:nL){

AKAK<-tcrossprod(diag(AKl1),diag(AKl1))

costheta<-AKl1*(AKAK^(−1/2))

costheta[which(costheta>1,arr.ind = TRUE)] = 1

theta<-acos(costheta)

AKl<-(1/pi)*(AKAK^(1/2))*(sin(theta)+(pi-theta)*cos(theta))

AKl1 = AKl

}

AKl<-AKl/median(AKl)

rownames(AKl)<-rownames(AK1)

colnames(AKl)<-colnames(AK1)

return(AKl)

}

########Exponencial Kernel############

K.exponential=function(x1,x2=x1, gamma=1){

exp(-gamma*outer(1:nrow(x1<- as.matrix(x1)), 1:ncol(x2<- t(x2)),

Vectorize(function(i, j) l2norm(x1[i,]-x2[,j]))))}

if (name==“Linear”) {

K=X%*%t(X)/p

} else if (name==“Polynomial”) {

K=K.Polynomial(x1=x1, x2=x1, gamma=1/p, b=0, d=d)

} else if (name==“Sigmoid”) {

K=K.Sigmoid(x1=x1, x2=x1, gamma=1/p, b=0)

}else if (name==“Gaussian”) {

K=K.Gaussian(x1=x1, x2=x1, gamma=1/p)

} else if (name==“AK1”) {

K= K.AK1_Final(x1=x1, x2=x1)

} else if (name==“AKL”) {

AK1=K.AK1_Final(x1=x1, x2=x1)

K=AK_L_Final(AK1=AK1,nL=nL)

} else {

K=K.exponential(x1=x1,x2=x1,gamma=1/p)

}

}

## Appendix B. Computation of kernels using the function given in Appendix A

rm(list=ls())

library(BGLR)

library(BMTME)

load(‘Data_Wheat_2019.RData’,verbose=TRUE)

ls()

#Phenotypic data

Markers=scale(Markers_Toy)

head(Markers)

dim(Markers)

colnames(Markers)=paste0(“M”,1:29157)

XF=round(Markers[1:6,1:5],3)

XF

source(“Kernel_construction.R”)

#Marker data

K.Linear=Kernel_computation(X=XF,name=“Linear”, degree=NULL, nL=NULL)

round(K.Linear,3)

K.Poly=Kernel_computation(X=XF,name=“Polynomial”, degree=2,nL=NULL)

round(K.Poly,3)

K.Sigmoid=Kernel_computation(X=XF,name=“Sigmoid”, degree=NULL,nL=NULL)

round(K.Sigmoid,3)

K.Gaussian=Kernel_computation(X=XF,name=“Gaussian”, degree=NULL,nL=NULL)

round(K.Gaussian,3)

K.AK1=Kernel_computation(X=XF,name=“AK1”, degree=NULL,nL=NULL)

round(K.AK1,3)

K.AK2=Kernel_computation(X=XF,name=“AKL”, degree=NULL,nL=2)

round(K.AK2,3)

K.AK15=Kernel_computation(X=XF,name=“AKL”, degree=NULL,nL=15)

round(K.AK15,3)

K.Exp=Kernel_computation(X=XF,name=“exponential”,degree=NULL,nL=NULL)

round(K.Exp,3)

## Appendix C. R code for the single environment under 7 kernels with data Data_Wheat_2019.RData. Results of Table 1

rm(list=ls())

library(BGLR)

library(BMTME)

load(‘Data_Wheat_2019.RData’,verbose=TRUE)

ls()

#Phenotypic data

Markers=scale(Markers_Toy)

head(Markers)

dim(Markers)

colnames(Markers)=paste0(“M”,1:29157)

XF=Markers[,colSums(!is.na(Markers)) > 0]

dim(XF)

source(“Kernel_construction.R”)

kernel_name=c(“Linear”,“Polynomial”, “Sigmoid”, “Gaussian”, “AK1”, “AKL”,“Exponential”)

K=Kernel_computation(X=XF,name=kernel_name[1], degree=2,nL=5)

head(K[1:10,1:10])

unique(Pheno_Toy$Env)

Pheno_JBL=Pheno_Toy[Pheno_Toy$Env==“JBL”,]

row.names(Pheno_JBL)=1:nrow(Pheno_JBL)

dim(Pheno_JBL)

head(Pheno_JBL)

#Marker data

#####design matrix of lines

Z_L=model.matrix(~0+GID,data=Pheno_JBL)

dim(Z_L)

round(K.Exp,3)

n=dim(Pheno_JBL)[1]

y=Pheno_JBL$DTHD

#Number of random partitions

K=10

set.seed(1)

PT = replicate(K,sample(n,0.20*n))

kernel_name=c(“Linear”,“Polynomial”, “Sigmoid”, “Gaussian”, “AK1”, “AKL”,“Exponential”)

results_all_kernels=data.frame()

results_all=data.frame()

for (i in 1:7) {

# i=1

K_Lines=Kernel_computation(X=XF,name=kernel_name[i], degree=2,nL=5)

K_expanded=Z_L%*%K_Lines%*%t(Z_L)

ETA=list(list(model=‘RKHS’,K=K_expanded))

Tab1_Metrics= data.frame(PT = 1:K,MSE = NA)

start_time<- proc.time()

for(k in 1:K) {

Pos_tst =PT[,k]

y_NA = y

y_NA[Pos_tst] = NA

A = BGLR(y=y_NA,ETA=ETA,nIter = 1e4,burnIn = 1e3,verbose = FALSE)

yp_ts = A$yHat

Tab1_Metrics$MSE[k] = mean((y[Pos_tst]-yp_ts[Pos_tst])^2)

Tab1_Metrics$Cor[k] = cor(y[Pos_tst],yp_ts[Pos_tst])

}

end_time<- proc.time()

Time=c(end_time[1] - start_time[1])

Metrics=apply(Tab1_Metrics[,-c(1)],2,mean)

Metrics_SE=apply(Tab1_Metrics[,-c(1)],2,sd)/sqrt(K)

results_all=rbind(results_all,data.frame(kernel=kernel_name[i],MSE=Metrics[1],SE_MSE=Metrics_SE[1],Cor=Metrics[2], SE_Cor=Metrics_SE[2], Time=Time))

}

results_all

write.csv(results_all,file=“results_kernels_single_environment_analysis_Table1.csv”)

## Appendix D. R code for the multi-environment analysis under 7 kernels with data Data_Wheat_2019.RData. Results of Table 2

rm(list=ls())

library(BGLR)

library(BMTME)

load(‘Data_Wheat_2019.RData’,verbose=TRUE)

ls()

#Phenotypic data

Markers=scale(Markers_Toy)

dim(Markers)

colnames(Markers)=paste0(“M”,1:29157)

XF=Markers[,colSums(!is.na(Markers)) > 0]

source(“Kernel_construction.R”)

kernel_name=c(“Linear”,“Polynomial”, “Sigmoid”, “Gaussian”, “AK1”, “AKL”,“Exponential”)

K=Kernel_computation(X=XF,name=kernel_name[1], degree=2,nL=5)

head(K[1:10,1:10])

unique(Pheno_Toy$Env)

Pheno=Pheno_Toy

row.names(Pheno)=1:nrow(Pheno)

#####design matrix of lines

Z_L=model.matrix(~0+GID,data=Pheno)

Z_E=model.matrix(~0+Env,data=Pheno)

K.E=Z_E%*%t(Z_E)

n=dim(Pheno)[1]

y=Pheno$DTHD

#Number of random partitions

K=10

set.seed(1)

PT = replicate(K,sample(n,0.20*n))

kernel_name=c(“Linear”,“Polynomial”, “Sigmoid”, “Gaussian”, “AK1”, “AKL”,“Exponential”)

results_all_kernels=data.frame()

results_all=data.frame()

for (i in 1:7) {

# i=1

K_Lines=Kernel_computation(X=XF,name=kernel_name[i], degree=2,nL=5)

K_expanded=Z_L%*%K_Lines%*%t(Z_L)

K_GE=K_expanded*K.E

ETA=list(Env=list(model=‘FIXED’,X=Z_E),Lines=list(model=‘RKHS’,K=K_expanded),GE=list(model=‘RKHS’,K=K_GE))

Tab1_Metrics= data.frame(PT = 1:K,MSE = NA)

start_time<- proc.time()

for(k in 1:K) {

Pos_tst =PT[,k]

y_NA = y

y_NA[Pos_tst] = NA

A = BGLR(y=y_NA,ETA=ETA,nIter = 1e4,burnIn = 1e3,verbose = FALSE)

yp_ts = A$yHat

Tab1_Metrics$MSE[k] = mean((y[Pos_tst]-yp_ts[Pos_tst])^2)

Tab1_Metrics$Cor[k] = cor(y[Pos_tst],yp_ts[Pos_tst])

}

end_time<- proc.time()

Time=c(end_time[1] - start_time[1])

Metrics=apply(Tab1_Metrics[,-c(1)],2,mean)

Metrics_SE=apply(Tab1_Metrics[,-c(1)],2,sd)/sqrt(K)

results_all=rbind(results_all,data.frame(kernel=kernel_name[i],MSE=Metrics[1],SE_MSE=Metrics_SE[1],Cor=Metrics[2], SE_Cor=Metrics_SE[2], Time=Time))

}

results_all

write.csv(results_all,file=“results_kernels_multi_environment_analysis_Table2.csv”)

## Appendix E. R code for the multi-trait multi-environment analysis under seven kernels with data Data_Wheat_2019.RData. Results found in Table 3

rm(list=ls())

library(BGLR)

library(BMTME)

library(plyr)

library(tidyr)

library(dplyr)

load(‘Data_Wheat_2019.RData’,verbose=TRUE)

ls()

#Phenotypic data

Markers=scale(Markers_Toy)

dim(Markers)

colnames(Markers)=paste0(“M”,1:29157)

XF=Markers[,colSums(!is.na(Markers)) > 0]

dim(XF)

source(“Kernel_construction.R”)

kernel_name=c(“Linear”,“Polynomial”, “Sigmoid”, “Gaussian”, “AK1”, “AKL”,“Exponential”)

K=Kernel_computation(X=XF,name=kernel_name[1], degree=2,nL=5)

head(K[1:10,1:10])

unique(Pheno_Toy$Env)

Pheno=Pheno_Toy

row.names(Pheno)=1:nrow(Pheno)

dim(Pheno)

head(Pheno)

#####design matrix of lines

Z_L=model.matrix(~0+GID,data=Pheno)

dim(Z_L)

Z_E=model.matrix(~0+Env,data=Pheno)

dim(Z_E)

K.E=Z_E%*%t(Z_E)

round(K.Exp,3)

n=dim(Pheno)[1]

y=Pheno[,4:5]

head(y)

head(Pheno[,1:6])

#Number of random partitions

K=10

set.seed(1)

PT = replicate(K,sample(n,0.20*n))

kernel_name=c(“Linear”,“Polynomial”, “Sigmoid”, “Gaussian”, “AK1”, “AKL”,“Exponential”)

results_all_kernels=data.frame()

results_all=data.frame()

source(‘PC_MM.R’)#See below

for (i in 1:7) {

# i=1

K_Lines=Kernel_computation(X=XF,name=kernel_name[i], degree=2,nL=5)

K_expanded=Z_L%*%K_Lines%*%t(Z_L)

K_GE=K_expanded*K.E

ETA=list(Env=list(model=‘FIXED’,X=Z_E),Lines=list(model=‘RKHS’,K=K_expanded),GE=list(model=‘RKHS’,K=K_GE))

Tab1_Metrics= data.frame()

start_time<- proc.time()

for(k in 1:K) {

#k=1

Pos_tst =PT[,k]

y_NA = data.matrix(y)

y_NA[Pos_tst,] = NA

A1= Multitrait(y = y_NA, ETA=ETA,resCov = list(type =“UN”, S0=diag(2),df0= 5),

nIter =10000, burnIn = 1000)

Metrics= PC_MM_f(y[Pos_tst,],A1$ETAHat[Pos_tst,],Env=Pheno$Env[Pos_tst])

Metrics

Tab1_Metrics=rbind(Tab1_Metrics, data.frame(Fold=k,Trait=Metrics[,1],Env=Metrics[,2],MSE=Metrics[,4],Cor=Metrics[,3]))

Tab1_Metrics

}

Tab1_Metrics

Summary<- Tab1_Metrics %>%group_by(Trait,Env) %>%

summarise(SE_MSE=sd(MSE, na.rm = T)/sqrt(n()),MSE=mean(MSE),SE_Cor = sd(Cor, na.rm = T)/sqrt(n()),Cor=mean(Cor))

results_all=rbind(results_all,data.frame(kernel=kernel_name[i],Summary))

results_all

}

SummaryT<- results_all %>%group_by(kernel,Trait) %>%

summarise(MSE=mean(MSE),SE_MSE=mean(SE_MSE),Cor=mean(Cor),SE_Cor =mean(SE_Cor))

Tab_R = as.data.frame(SummaryT)

Tab_R

write.csv(Tab_R,file=“results_kernels_multitrait_Table3.csv”)

## Appendix F. R code for the multi-environment analysis with ordinal response variable under seven kernels with data Data_Wheat_2019.RData. Results found in Table 4

rm(list=ls())

library(BGLR)

library(BMTME)

library(caret)

load(‘Data_Wheat_2019.RData’,verbose=TRUE)

#Phenotypic data

Markers=scale(Markers_Toy)

colnames(Markers)=paste0(“M”,1:29157)

XF=Markers[,colSums(!is.na(Markers)) > 0]

source(“Kernel_construction.R”)

kernel_name=c(“Linear”,“Polynomial”, “Sigmoid”, “Gaussian”, “AK1”, “AKL”,“Exponential”)

K=Kernel_computation(X=XF,name=kernel_name[1], degree=2,nL=5)

Pheno=Pheno_Toy

row.names(Pheno)=1:nrow(Pheno)

#####design matrix of lines

Z_L=model.matrix(~0+GID,data=Pheno)

Z_E=model.matrix(~0+Env,data=Pheno)

K.E=Z_E%*%t(Z_E)

n=dim(Pheno)[1]

y=Pheno$DTHD

pos_JBL=which(Pheno_Toy$Env==“JBL”)

summary(y[pos_JBL])

y[pos_JBL]=ifelse(y[pos_JBL]<78.09,1,ifelse(y[pos_JBL]>80.95,3, 2))

y[pos_JBL]

pos_LDH=which(Pheno_Toy$Env==“LDH”)

y[pos_LDH]=ifelse(y[pos_LDH]<102.20,1,ifelse(y[pos_LDH]>105.99,3, 2))

y[pos_LDH]

pos_PUS=which(Pheno_Toy$Env==“PUS”)

y[pos_PUS]=ifelse(y[pos_PUS]<82.97,1,ifelse(y[pos_PUS]>89.48,3, 2))

y[pos_PUS]

#Number of random partitions

K=10

set.seed(1)

PT = replicate(K,sample(n,0.20*n))

kernel_name=c(“Linear”,“Polynomial”, “Sigmoid”, “Gaussian”, “AK1”, “AKL”,“Exponential”)

results_all_kernels=data.frame()

results_all=data.frame()

for (i in 1:7) {

K_Lines=Kernel_computation(X=XF,name=kernel_name[i], degree=2,nL=5)

K_expanded=Z_L%*%K_Lines%*%t(Z_L)

K_GE=K_expanded*K.E

ETA=list(Env=list(model=‘FIXED’,X=Z_E),Lines=list(model=‘RKHS’,K=K_expanded),GE=list(model=‘RKHS’,K=K_GE))

Tab1_Metrics= data.frame(PT = 1:K,PCCC = NA, Kappa=NA)

start_time<- proc.time()

for(k in 1:K) {

Pos_tst =PT[,k]

y_NA = y

y_NA[Pos_tst] = NA

A = BGLR(y=y_NA,ETA=ETA,response_type=“ordinal”, nIter = 1e4,burnIn = 1e3,verbose = FALSE)

Probs = A$probs[Pos_tst,]

yp_ts = apply(Probs,1,which.max)

CM=confusionMatrix(as.factor(yp_ts),as.factor(y[Pos_tst]))

Tab1_Metrics$PCCC[k] = 1-mean(y[Pos_tst]!=yp_ts)

Tab1_Metrics$Kappa[k]=c(CM$overall[2])

}

end_time<- proc.time()

Time=c(end_time[1] - start_time[1])

Metrics=apply(Tab1_Metrics[,-c(1)],2,mean)

Metrics_SE=apply(Tab1_Metrics[,-c(1)],2,sd)/sqrt(K)

results_all=rbind(results_all,data.frame(kernel=kernel_name[i],PCC=Metrics[1],SE_PCCC=Metrics_SE[1],Kappa=Metrics[2], SE_Kappa=Metrics_SE[2], Time=Time))

}

results_all

write.csv(results_all,file=“results_kernels_multi_environment_analysis_Ordinal_Table4.csv”)

## Appendix G. Code for computing sparse kernels

Sparse_Kernel_Construction=function(m,X,name,degree,nL){

degree=degree

nl=nL

m=m

XF=X

p=ncol(XF)

pos_m=sample(1:nrow(XF),m)

X_m=XF[pos_m,]

dim(X_m)

########Gaussian Kernel function############

l2norm=function(x){sqrt(sum(x^2))}

K.radial=function(x1,x2=x1, gamma=1){

exp(-gamma*outer(1:nrow(x1<- as.matrix(x1)), 1:ncol(x2<- t(x2)),

Vectorize(function(i, j) l2norm(x1[i,]-x2[,j])^2)))}

########Polynomial Kernel############

K.polynomial=function(x1, x2=x1, gamma=1, b=0, d=degree)

{ (gamma*(as.matrix(x1)%*%t(x2))+b)^d}

########Sigmoid Kernel############

K.sigmoid=function(x1,x2=x1, gamma=1, b=0)

{ tanh(gamma*(as.matrix(x1)%*%t(x2))+b) }

########Exponencial Kernel############

K.exponential=function(x1,x2=x1, gamma=1){

exp(-gamma*outer(1:nrow(x1<- as.matrix(x1)), 1:ncol(x2<- t(x2)),

Vectorize(function(i, j) l2norm(x1[i,]-x2[,j]))))}

############Arcosine kernel with deep=1#######

K.AK1_Final<-function(x1,x2){

n1<-nrow(x1)

n2<-nrow(x2)

x1tx2<-x1%*%t(x2)

norm1<-sqrt(apply(x1,1,function(x) crossprod(x)))

norm2<-sqrt(apply(x2,1,function(x) crossprod(x)))

costheta = diag(1/norm1)%*%x1tx2%*%diag(1/norm2)

costheta[which(abs(costheta)>1,arr.ind = TRUE)] = 1

theta<-acos(costheta)

normx1×2<-norm1%*%t(norm2)

J = (sin(theta)+(pi-theta)*cos(theta))

AK1 = 1/pi*normx1×2*J

AK1<-AK1/median(AK1)

colnames(AK1)<-rownames(x2)

rownames(AK1)<-rownames(x1)

return(AK1)

}

####Kernel Arc-Cosine with deep=L#####

diagAK_f<-function(dAK1)

{

AKAK = dAK1^2

costheta = dAK1*AKAK^(−1/2)

costheta[which(costheta>1,arr.ind = TRUE)] = 1

theta = acos(costheta)

AKl = (1/pi)*(AKAK^(1/2))*(sin(theta)+(pi-theta)*cos(theta))

AKl

AKl<-AKl/median(AKl)

}

AK_L_Final<-function(AK1,dAK1,nl){

n1<-nrow(AK1)

n2<-ncol(AK1)

AKl1 = AK1

for (l in 1:nl){

AKAK<-tcrossprod(dAK1,diag(AKl1))

costheta<-AKl1*(AKAK^(−1/2))

costheta[which(costheta>1,arr.ind = TRUE)] = 1

theta<-acos(costheta)

AKl<-(1/pi)*(AKAK^(1/2))*(sin(theta)+(pi-theta)*cos(theta))

dAKl = diagAK_f(dAK1)

AKl1 = AKl

dAK1 = dAKl

}

AKl<-AKl/median(AKl)

rownames(AKl)<-rownames(AK1)

colnames(AKl)<-colnames(AK1)

return(AKl)

}

AK_ALL=K.AK1_Final(x1=XF,x2=XF)

AK11=K.AK1_Final(x1=XF,x2=X_m)

AKL2=AK_L_Final(AK1=AK11,dAK1=diag(AK_ALL),nl=5)

dim(AKL2)

####Step 1 compute K_m###############

if (name==“Linear”) {

K_m=X_m%*%t(X_m)/p

######Step 2 comput K_n_m###########

K_n_m=XF%*%t(X_m)/p

} else if (name==“Polynomial”) {

K_m=K.polynomial(x1=X_m,x2=X_m,gamma=1/p)

######Step 2 comput K_n_m###########

K_n_m=K.polynomial(x1=XF,x2=X_m,gamma=1/p)

} else if (name==“Sigmoid”) {

K_m=K.sigmoid(x1=X_m,x2=X_m,gamma=1/p)

######Step 2 comput K_n_m###########

K_n_m=K.sigmoid(x1=XF,x2=X_m,gamma=1/p)

}else if (name==“Gaussian”) {

K_m=K.radial(x1=X_m,x2=X_m,gamma=1/p)

######Step 2 comput K_n_m###########

K_n_m=K.radial(x1=XF,x2=X_m,gamma=1/p)

} else if (name==“AK”) {

K_m=K.AK1_Final(x1=X_m,x2=X_m)

######Step 2 comput K_n_m###########

K_nm1=K.AK1_Final(x1=XF,x2=X_m)

K_all=K.AK1_Final(x1=XF,x2=XF)

K_n_m=AK_L_Final(AK1=K_nm1,dAK1=diag(K_all),nl=nl)

} else {

K_m=K.exponential(x1=X_m,x2=X_m,gamma=1/p)

######Step 2 comput K_n_m###########

K_n_m=K.exponential(x1=XF,x2=X_m,gamma=1/p)

}

######Step 3 compute Eigen value decomposition of K_m######

EVD_K_m=eigen(K_m)

####Eigenvectors

U=EVD_K_m$vectors

###Eigenvalues###

S=EVD_K_m$values

S[which(S<0)]=0

####Square root of the inverse of eigenvelues #####

S_0.5_Inv=sqrt(1/S)

#####Diagonal matrix of square root of inverse of ingenvalues###

S_mat_Inv=diag(S_0.5_Inv)

######Computing matrix P

P=K_n_m%*%U%*%S_mat_Inv

return(P)}

## Appendix H. Illustration of the computation of the design matrix to approximate kernels

m(list=ls())

library(BGLR)

library(BMTME)

load(‘Data_Wheat_2019.RData’,verbose=TRUE)

ls()

#Phenotypic data

Markers=scale(Markers_Toy)

colnames(Markers)=paste0(“M”,1:29157)

var=apply(Markers,2,var)

pos_No_0=which(var>0)

Markers=Markers[,pos_No_0]

XF=round(Markers[1:10,],3)

dim(XF)

source(“Sparse_Kernel_Construction.R”)

K.Gaussian1=Sparse_Kernel_Construction(m=3,X=XF,name=“Gaussian”, degree=NULL,nL=NULL)

round(K.Gaussian1,3)

K.Gaussian2=Sparse_Kernel_Construction(m=5,X=XF,name=“Gaussian”, degree=NULL,nL=NULL)

round(K.Gaussian2,3)

K.Gaussian3=Sparse_Kernel_Construction(m=7,X=XF,name=“Gaussian”, degree=NULL,nL=NULL)

round(K.Gaussian3,3)

########Computation of P for other kernels######

kernel_name=c(“Linear”,“Polynomial”, “Sigmoid”, “Gaussian”, “AK1”, “AKL”,“Exponential”)

K.AKL=Sparse_Kernel_Construction(m=7,X=XF,name=kernel_name[6], degree=NULL,nL=5)

round(K.AKL,3)

## Appendix I. Example that illustrate the implementation of approximate kernels under seven kernels

rm(list=ls())

library(BGLR)

library(BMTME)

load(‘Data_Toy_EYT.RData’,verbose=TRUE)

ls()

XF=t(chol(G_Toy_EYT))

source(“Sparse_Kernel_Construction_Appendix_G.R”)

Pheno=Pheno_Toy_EYT

row.names(Pheno)=1:nrow(Pheno)

#####design matrix of lines

Z_L=model.matrix(~0+GID,data=Pheno)

Z_E=model.matrix(~0+Env,data=Pheno)

n=dim(Pheno)[1]

y=Pheno$GY

head(Pheno)

#Number of random partitions

K=10

set.seed(3)

PT = replicate(K,sample(n,0.10*n))

kernel_name=c(“Linear”,“Polynomial”, “Sigmoid”, “Gaussian”, “AK1”, “AKL”,“Exponential”)

results_all_m=data.frame()

mvalues=c(10, 15, 20, 35, 40)

for (s in 1:5){

results_all=data.frame()

for (i in 1:7) {

P_Lines=Sparse_Kernel_Construction(m=mvalues[s],X=XF,name=kernel_name[i], degree=2,nL=5)

Var0=apply(P_Lines,2,sd)

pos_varNo0=which(Var0>0)

P_Lines1=P_Lines[,pos_varNo0]

P_expanded=Z_L%*%P_Lines1

P_GE=model.matrix(~0+P_expanded:Env,data=Pheno)

ETA=list(Env=list(model=‘FIXED’,X=Z_E),Lines=list(model=‘BRR’,X=P_expanded),GE=list(model=‘BRR’,X=P_GE))

Tab1_Metrics= data.frame(PT = 1:K,MSE = NA)

start_time<- proc.time()

for(k in 1:K) {

Pos_tst =PT[,k]

y_NA = y

y_NA[Pos_tst] = NA

A = BGLR(y=y_NA,ETA=ETA,nIter = 1e4,burnIn = 1e3,verbose = FALSE)

yp_ts = A$yHat

Tab1_Metrics$MSE[k] = mean((y[Pos_tst]-yp_ts[Pos_tst])^2)

Tab1_Metrics$Cor[k] = cor(y[Pos_tst],yp_ts[Pos_tst])

}

end_time<- proc.time()

Time=c(end_time[1] - start_time[1])

Metrics=apply(Tab1_Metrics[,-c(1)],2,mean)

Metrics_SE=apply(Tab1_Metrics[,-c(1)],2,sd)/sqrt(K)

results_all=rbind(results_all,data.frame(kernel=kernel_name[i],MSE=Metrics[1],SE_MSE=Metrics_SE[1],Cor=Metrics[2], SE_Cor=Metrics_SE[2], Time=Time))

}

results_all

results_all_m=rbind(results_all_m,data.frame(m=mvalues[s],results_all))

}

results_all_m

write.csv(results_all_m,file=“results_Sparse_kernels_multi_environment_analysis_Table5.csv”)
